# Brain donation rules in Italy and worldwide: overview of a cutting-edge topic for human brain research

**DOI:** 10.1007/s10072-025-08214-7

**Published:** 2025-05-06

**Authors:** Vittorio Bolcato, Tino Emanuele Poloni, Giuseppe Basile, Annalisa Davin, Riccardo Rocco Ferrari, Giulia Negro, Arcangelo Ceretti, Antonio Guaita, Livio Pietro Tronconi

**Affiliations:** 1 Astolfi Associates Legal Firm, Milan & Maria Beatrice Hospital, GVM Care and Research, Florence, Italy; 2 Department of Neurology and Neuropathology, Golgi Cenci Foundation, Abbiategrasso, MI Italy; 3 Department of Rehabilitation, ASP Golgi Redaelli, Abbiategrasso, MI Italy; 4 Golgi Cenci Foundation, Laboratory of Neurobiology and Neurogenetics, Abbiategrasso, MI Italy; 5 Department of Biomedical Sciences and Public Health, University “Politecnica Delle Marche” of Ancona, Ancona, AN Italy; 6 Department of Life and Human Sciences, European University of Rome, Rome, Italy; 7 Scientific Directorate, GVM Care and Research, Maria Cecilia Hospital, Cotignola, RA Italy

**Keywords:** Neuroscience, Brain donation, Body donation, Health regulations, Brain bank, *Post-mortem* interval

## Abstract

Neuropathological examination of the brain and its biochemical analyses are fundamental to neuroscience studies and public health decisions, but are dependent on the effectiveness of regulations and operational protocols. The article discusses opportunities and limits of Italian regulation on body donation in relation to the specific requirements of neuropathology and brain sciences, in comparison with the regulations of other countries. Some crucial issues emerge, widely shared in the various regulatory contexts. The main aspect is the willingness to donate, consciously expressed by the subject by signing an informed consent or through the formulation of advanced directives. The donation of a single organ, the brain in particular, does not necessarily imply the donation of the entire body, which should be considered separately. In the specific case of the brain, particular attention is given to reducing the *post-mortem* interval, in order to obtain tissues suitable for research. Consequently, the centres that deal with the brain and brain banking must have experience and expertise in handling nervous tissue, and do not necessarily have to deal with the management of the entire body. These aspects, still little addressed in Italy, are the basis to develop an effective brain banking activity, which can only develop by integrating *post-mortem* body donation with specific rules for brain banking without which Italian neuroscience will be penalised in the coming years.

## Introduction

Considering the epidemiological explosion of age-related neurodegenerative diseases, the study of neurodegeneration and brain diseases constitutes a scientific breakthrough and an important public health goal. Because animal models cannot fully explain human pathophysiology [1], a comprehensive investigation of human neurodegeneration necessarily relies on *post-mortem* Brain Donation (pBD) [2]. Histopathological characterization and *post-mortem* biochemical analyses on frozen brain tissue are necessary to develop"precision neurology", which integrates clinicopathological correlations and omics-based approaches to unravel the etiopathogenesis of neurodegenerative diseases. This approach is the basis for searching and validating biological markers for early diagnosis and identification of specific therapeutic targets that lead to personalized therapies [3–5]. For these purposes, it is crucial to consider the rapid *post-mortem* brain deterioration, which progressively alters tissue architecture and its biochemical characteristics. Indeed, as the *post-mortem* interval increases, biological information is progressively lost [6, 7], including reduction in synaptic density [8], and RNA deterioration [9]. Therefore, the brain *Post-Mortem* Interval (bPMI—time between death and brain explant, harvesting and processing) should be minimized, ideally remaining below 24 h [10, 11]. Globally, this is possible through the combination of pBD programs with efficient brain banking protocols [4, 12]. Several brain banks conduct brain extraction within a few hours [13, 14]. For instance, the Sun Health Research Institute (Sun City, USA) has a median bPMI of 2.7 h [15], and the Netherlands and Australian Brain Banks between 4 and 12 h [16]. Noteworthy, Australian Mark Hughes Foundation Brain Biobank manages to have bPMI with a maximum of 12 h in some rural areas with poor healthcare provisions, through planning, networking and mortuary service organisation [17].

Since 2009, the first Italian pBD program has been developed as part of a cohort study conducted in the town of Abbiategrasso (“InveCe.Ab” — brain aging in Abbiategrasso, Milan), which obtained ethical approval to perform brain donation [18]. This project has demonstrated the feasibility of brain banking in Italy. Nevertheless, the InveCe.Ab study is nearing its conclusion, and current Italian law does not favor the continuation of pBD programs. Therefore, it is imperative to propose solutions to promote long-lasting pBD programs in Italy. Recently, Cattaneo et al. conducted a nation-wide survey to investigate public attitudes and concerns surrounding pBD [19]. For the first time, this manuscript addresses the challenges posed by pBD in Italy, offering only preliminary insights into potential solutions. Starting from our expertise and the issues raised by the aforementioned study, we address the topic of pBD through a comprehensive review of the Italian regulatory context. To overcome the obstacles to pBD, we propose strategies to optimize the regulation and minimize the bPMI, while respecting donor intentions in relation to the laws in force in Italy and brain banking organization.

## References and regulations in Italy

### Regulations on the determination of cardiac and brain death and mortuary police activities

Two decrees (**decree of President of the Republic, 10 September 1990, N. 285; decree of the Health Ministry, 11 April 2008—update of decree 22 August 1994 N. 582**), and a law of the Italian Parliament (**Law 29 December 1993, N. 578**) contain the rules about death ascertainment and mortuary police activities [20–22]. From the combined reading of the national and regional regulations, first comes the definition of death, which is the “irreversible cessation of all the functions of the brain”. This condition may be secondary to cardiac arrest (*cardiac* death) or due to extensive damage to the brain (*brain* death). As a rule, death is ascertained based on *cardiac* arrest. Indeed, an irreversible and prolonged cardio-respiratory arrest reliably indicates the irreversible cessation of all brain functions. Confirmation of death is achieved either by the observation of consecutive abiotic signs, over a period of 15- and 30-h *post-mortem* or by instrumental ascertainment through a flat electrocardiogram (ECG) for 20 consecutive minutes (*i.e.* thanatography) [23]. Only after this confirmation and its certification, the body is eligible for manipulation (e.g., brain extraction, harvesting, transplantation, or funeral). On the other hand, patients with extensive brain damage may temporarily preserve cardiac activity; *brain* death diagnosis is more complex, as it requires repeated clinical and neurophysiological tests to confirm the persistent absence of brain function. While these patients can donate organs, they are evidently ineligible to brain donation.

Acquiring the brain promptly after death is feasible with an efficient organization, as it would be sufficient to demonstrate the absence of cardiac activity through 20-min thanatography, and then proceed with the brain explant and processing to avoid the rapid deterioration of brain tissue. There may be differences depending on the circumstances of death. In cases of death in a healthcare facility, an appointed physician performs the 20-min thanatography and confirms death, thus making the body available for a timely brain extraction. In non-healthcare settings, death ascertainment should be performed through thanatography at the biobank or other dissection rooms, following pre-established protocols to transfer the body, thereby saving time and preserving tissues. In Italy, thanatography is already implemented within healthcare facilities for corneal transplantation through a specific regulation (**Law 12 August 1993, n. 301 Rules on corneal sampling and transplantation**). This legislation was specifically designated to facilitate a properly corneal donation. In such cases, the corneas taking is performed at the hospital morgue after timely death ascertainment through thanatography. A similar approach could be applied to pBD, enabling prompt brain retrieval.

Any biological material, including the brain, must be harvested and managed by a biobank, which guarantees the systematic collection and management of disease-oriented or population-based biological samples [12, 24–26]. Biobanks are developing significantly, even in Italy, with therapeutic purposes and, more recently, also research purposes. Particularly, research biobanks are aimed at collection, storage and distribution of human biological material and related data in a traceable and secure manner for scientific research purpose, guaranteeing the rights of the subjects involved through informed consent [27, 28]. The Biobanking and Biomolecular Resources Research Infrastructure is a European Research Infrastructure (BBMRI—ERIC) comprising biobanks and biomolecular resources. It operates in accordance with quality standards and the Ethical, Legal, and Social Implications (ELSI) requirements, and it offers the geographical distribution of the main biobanks worldwide [29, 30].

### Provisions on organ and tissue harvesting and transplantation

The **Law 1° April 1999, n. 91 about organ donation** is based on an earlier model of presumed consent, or opt-in model (tacit acceptance), with the possibility of family opposition [31]. This setting is superseded by the **Law 22 December 2017, n. 219 on informed consent and advance treatment provisions**, which regulates doctor-patient information, entitled person traceable consenting, and the establishment of advanced directives (ADs) for future healthcare wills [32]. This law is oriented toward a conscious, autonomous, and expressed will of the entitled person [33, 34]. Family members may participate, and support entitled subjects, but have no legal authority. A trustee is appointed to guarantee the ADs are followed; these are filed with a notary or privately and registered at the local health authority. The ADs should be easily accessible to physicians on the Ministry of Health gateway via digital identity, even if a recent regulatory Decree seems to limit the access [35]. A further development is due to the **Law 10 February 2020 n. 10 of Directives of own body and *post-mortem* tissues for the purposes of study, training and scientific research** and implementing decrees, after specific living ADs (article 3) [36]. The law also includes the identification of reference structures (article 4) through the definition of the criteria and minimum requirements of personnel, structures and organization [37]. The rule regulates the procedures for managing the entire body after death for scientific and didactic purposes, based on death ascertainment and regulations according to mentioned Mortuary Police Regulations [38]. Therefore, once death has been instrumentally ascertained and certified, no additional observation period is required, also because prompt conservation of the body is necessary [31, 39]. However, the purpose that eventually led to the Law 10/2020 was not the study of individual organs nor the advancement of neuroscience through the study of the brain. Rather, this law was conceived to allow and foster anatomical dissection and surgical training, in accordance with the individual’s expressed will. For this aim, the *post-mortem* interval is not crucial, as it does not determine significant macroscopic alterations of the body [40]. Nevertheless, these activities are still limited, due to the negative emotional impact associated with this type of donation. Indeed, the body is likely to become aesthetically unpresentable and will need to be cremated before being returned to the family, which occurs several months later (no more than a year after death) [39, 41]. A relevant issue remains the difficulty of the family in accepting either the delay or the renunciation to traditional death rituals. Furthermore, only a few centers can store the bodies for up to a year, covering the costs of management and cremation. This approach allows the management of only a limited number of bodies and does not meet the requirements for brain banking purposes.

## World perspective on body and brain biobanking

Globally, the context of post-mortem body donation is heterogeneous. Many countries continue to face limited citizens participations and awareness, resulting in a poor availability of donated bodies. As previously noted, religious, emotional and cultural determinants associated with death and burial often act as barriers that limit body donation. Worldwide, unclaimed bodies are the main source for didactic anatomical dissection and surgical training [42]. In contrast, several European countries have currently introduced specific regulations, based on informed consent or advanced disposition for body donation [43]. In the USA and Canada, body donation programs ground on informed consent and specific regulating acts. Countries such as India, Bangladesh, Indonesia, Iran and Türkiye, seem to have similar challenges with unclaimed bodies. China, after the 2001"Shanghai Regulations on Body Donation”, has developed local legislative initiative regulating body donation [44]. Japan, Korea and Sri Lanka have successfully addressed donor bodies need through legally defined body donation programs [45].

Regardless of whole-body donation, the legislation of several countries considers the possibility of donating single organs. In some countries, including the UK, Australia, and the USA, donors are enrolled in dedicated programs for the donation of single specific organs, such as the brain. This is usually referred to as “*antemortem* tissue” when the consent to donation was expressed by the subject during his/her lifetime (in some contexts, consent may be given by relatives after the subject's death, unless he/she had clearly expressed his/her opposition). In these systems, the healthcare facility where death occurs is responsible for retrieving the brain in the morgue and arranging its transfer to the specialized brain bank centre [15]. Alternatively, brain retrieval may be coordinated through contact with proxies and transport to the reference centre for the explant and harvesting, according to predefined protocols [17, 46–48]. Brain banks typically receive already extracted brain tissue. These centres have a brain specificity and do not necessarily deal with the management of the body. Following brain donation, the body of the deceased may be returned to the family for the funeral, used for other organs transplantation or research, or subjected to dissection practices for training purposes. These actions are carried out strictly according to the informed consent signed by the donor and/or based on the conditions of the enrolment program, which may be pathology-oriented [49]. It is also important to consider the legal requirements governing the determination of death, particularly the no-touch period, which corresponds to the time during which it is not possible to act on the corpse. The no-touch period depends upon clinical observation of the cadaveric phenomena or instrumental demonstration of asystole, generally by means of thanatography that allows the observation period to be greatly shortened with a significant impact on bPMI. Across Europe, the no-touch time generally ranges between 5 and 10 min of asystole (*e.g.* Austria, Belgium, Czech Republic, France, Netherlands, UK, Spain, and Switzerland), whereas in Italy it is set at 20 min [23]. The timing of brain removal may be influenced by participation in specific protocolized programs, such as in cases of euthanasia, as occurs in the Netherlands or Australia. These possibilities introduce additional ethical and regulatory complexity [50, 51]. Regarding brain banking, the process is globally governed by informed consent, as subject consent is a fundamental requirement for tissue collection in the biobank. In Europe, several countries regulate brain banking and brain tissue donation within broader administrative and legislative instruments such as healthcare regulations, biomedical research guidelines, genetic data rules, and more recently, data protection rules [28, 52]. The informed consent takes place in the present but expresses a future will (ADs), with all the complexities due to changes that may occur between the time of the disposition and death [53]. For instance, the United Kingdom Human Tissue Act 2004 (and the 2006 Act for Scotland) regulates all tissue banks across England, Wales, and Northern Ireland. These acts replaced the earlier ‘lack of objection’ criteria established in the Human Tissue Act 1961 with a more explicit and ethically robust model based on informed consent [54]. Other examples include the Portuguese 2005 Act on personal genetic and health information, the French 2011 regulation, the Norwegian 2008 Act on medical and health research; the Lithuanian 2000 Law on the Ethics of Biomedical Research; the Spanish 2007 Law on Biomedical Research. Other countries have adopted specific biobank-focused legislative acts, such as the Icelandic Act on Biobanks no. 110/2000; the Estonian 2000 Human Genes Research Act, the Hungarian 2008 Parliamentary Act on the protection of human genetic data and the regulation of human genetic studies, research and biobanks, the Swedish 2002 Biobank Act; the Belgian 2007 Law on Biomedical Research [52]. Outside Europe, the Australian Coroners Act and Human Tissue Act regulates brain donation, with regional variations [17]. USA relies on the Health Insurance Portability and Accountability Act (HIPAA). Compliance with HIPAA regulations now requires that Institutional Review Boards oversee brain and tissue banking proposals. In Canada, regulation is provided by the Human Tissue Act, which is applied at the provincial level. In China, a combination of laws, including the Law on Human Organ Transplantation (2007), the Ethics Review System for Biomedical Research (2016), the Biosecurity Law (2020) and Regulations on the Administration of Human Genetic Resources of the People’s Republic of China (2019), collectively regulates the brain banking practices [55–57]. Some relevant examples of the rules governing death ascertainment, body donation and brain banking are summarized in Table 1.

**Table 1:** Global comparison of no-touch times and rules for body donation and brain banking

Country	Death ascertainment procedures	No-touch time (minutes)	Presence of rules for body donation	Presence of rules for brain banking
European Examples				
Italy	Clinical cardiocirculatory arrest and thanatography	20’	✓	**X** Only single-study protocols
France	Clinical cardiocirculatory arrest and thanatography	5’	✓	✓
Spain	Clinical cardiocirculatory arrest and thanatography	5’	**X**	✓
Germany	Clinical cardiocirculatory arrest and thanatography	5–10’	✓	**X** Only single-study protocols
The Netherlands	Clinical cardiocirculatory arrest	5’	✓ Even after euthanasia	✓
UK	Clinical cardiocirculatory arrest and thanatography	5’	✓	✓
Worldwide Examples				
USA	Clinical cardiocirculatory arrest and optional thanatography	2–5’	✓	✓
Australia	Clinical cardiocirculatory arrest and optional thanatography	5’	✓ Even after euthanasia	✓
China	Clinical cardiocirculatory arrest and optional thanatography	5’	✓ On a regional basis	Evolving

Overall, this review highlights some important and widely shared aspects. The most important aspect is the willingness to donate, consciously expressed by the subject through the signing of an informed consent or, in a more general regulatory context, through the formulation of ADs. The donation of a single organ, the brain in particular, does not necessarily imply the donation of the entire body, which should be considered separately. Furthermore, maximum attention is paid to reduce the bPMI as much as possible, in order to preserve the quality of the tissue and its scientific informativeness. Consequently, the centres that deal with the brain and brain banking must have a specific expertise in handling nervous tissue, but do not necessarily have to deal with the management of the entire body. These aspects, still little addressed in Italy, are the basis to develop an effective brain banking activity.

## Proposals to effectively regulate brain-banking activities in Italy

The informed expression of will is the linchpin of donation for research within the framework of ADs [39]. Therefore, it is necessary to clarify the difference between body donation for surgical training, organ transplantation, and tissue donation for research [19, 58, 59]. It is also pivotal to meet in person with potential donors and provide a thorough explanation of everything that pBD entails, including its high scientific and social value [60]. The issue of research on the brain tissue must be balanced with the necessity to respect socio-cultural interests in grieving and associated rituals, as surveyed by Cattaneo et al. [19]. This context presents a field of considerable ethical-social and medico-legal delicacy. The peculiarity of brain explant and harvesting offers the advantage of returning the body within a few hours, carefully recomposed and visible in an open coffin, thereby ensuring no delays in the regular funeral arrangements.

**Fig. 1:**
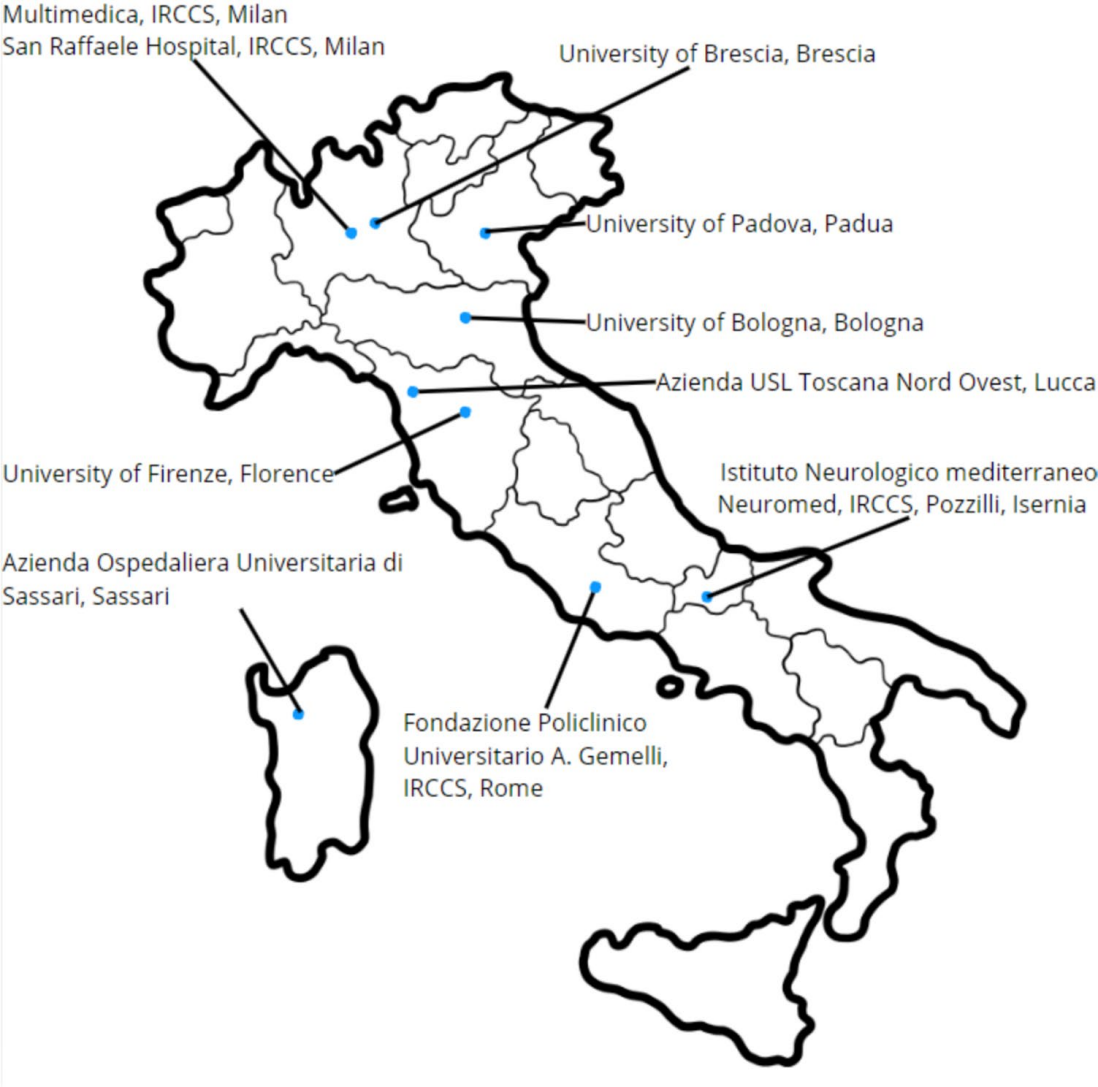
Italian distribution of Accredited Body Donation Centers

Considering that bPMI primarily depends on organizational protocols for body management [18], the question of dedicated neurological research centres arises. The notably uneven geographical distribution and varying research interests of the ten Italian reference centres currently recognized for body donation pose major issues to ensuring functional transfer and effective research (Fig. 1) [19]. In defining the requirements, the law considers only those facilities that can manage the body in its entirety, without mentioning the management of individual postmortem tissues, which is highly specific for brain. Hence, those requirements should not be applicable for pBD, as there are substantial differences: the brain removal process is more conservative, the body is quickly returned to the family for the funeral, and there is a pressing need to minimize bPMI as much as possible to obtain brains suitable for research. The amendment/integration by scope would not impact on the setup of the law 10/2020 nor of the mortuary regulations but would only answer to cogent specificities. The requirements for brain sampling and study entail distinct needs, perspectives and other requirements. All these factors should be considered; otherwise, an area of study of enormous scientific and social impact could be jeopardized. The current organizational framework presents no regulatory gaps; however, an enhancement in a domain of great social and scientific importance is necessary. The neuropathological field is extremely specific, both in terms of functional requirements and the peculiarities of the tissue. Therefore, it is necessary to refer to experienced centres, together with bioethics committee agreements, which are not exclusively related to *post-mortem* body donation. Furthermore, this approach could more effectively respond to specific wishes expressed in the ADs, as happens for families affected by neurodegenerative diseases [53]. It must also be considered that the subject may delineate limited areas of body donation [61].

## Conclusions

To promote pBD programs in Italy, it is necessary to act in three directions, as also suggested in the work of Cattaneo et al. First, it is essential to reach out individuals who might be part of a brain donation program by addressing their concerns, inquiries and expectations. Establishing structured networks to maintain ongoing relationships with brain donors, patients’ associations and citizens is vital for fostering a supportive and informed community. The second step involves completing the legislative framework to address and resolve specific challenges associated with specific organs tissue donation. It is important to recognize the huge difference between the brain, which deteriorates rapidly, and the entire body or other tissues, especially considering ADs arrangement and working protocols. The ADs model established under the Law 10/2020 may not provide a fully functional, respectful, and practical pathway for pBD and therefore require revisions. The third critical point is to establish organizational protocols that meet brain banks requirements, encompassing all activities related to collection, characterization, storage and distribution of brain material, ensuring its scientific and ethical use. The definition and identification of dedicated “Centres for Brain Research and Donation” will facilitate comparison and collaboration with European and International players, thereby ensuring Italy's effective entry into an international brain banking network. Without these regulatory and organizational adjustments, brain banking activities in Italy will not be eligible to enter a European network and will remain limited to individual studies and initiatives. In this context, neuropathological studies will be confined to a limited number of available subjects. If the issue is not addressed, it will cause a delay in Italian neuroscientific development.

## Data Availability

Not applicable.

## Abbreviations

*pBD*: *post-mortem* Brain Donation
*bPMI*: Brain *post-mortem* interval
*ADs*: Advanced directives
